# Double Trouble: Does Co-Morbid Chronic Somatic Illness Increase Risk for Recurrence in Depression? A Systematic Review

**DOI:** 10.1371/journal.pone.0057510

**Published:** 2013-03-05

**Authors:** Gemma D. Kok, Claudi L. H. Bockting, Huibert Burger, Wiebke Hannig, Gerdina H. M. Pijnenborg, Pim Cuijpers, Steven D. Hollon

**Affiliations:** 1 Department of Clinical Psychology, University of Groningen, Groningen, The Netherlands; 2 Department of General Practice, University Medical Center Groningen, University of Groningen, Groningen, The Netherlands; 3 Department of Clinical Psychology and Psychotherapy, Marburg University, Marburg, Germany; 4 Department of Psychotic Disorders, GGZ Drenthe, Assen, The Netherlands; 5 Department of Clinical Psychology and EMGO+ Institute for Health and Care Research, VU University and VU University Medical Centre, Amsterdam, The Netherlands; 6 Leuphana University, Lüneburg, Germany; 7 Department of Psychology, Vanderbilt University, Nashville, Tennessee, United States of America; Federal University of Rio de Janeiro, Brazil

## Abstract

**Objective:**

To perform a systematic review, and if possible a meta-analysis, to establish whether depressed patients with co-morbid chronic somatic illnesses are a high risk “double trouble” group for depressive recurrence.

**Method:**

The databases PubMed, EMbase and PsycINFO were systematically searched until the 4^th^ of December 2012 by using MeSH and free text terms. Additionally, reference lists of retrieved publications and treatment guidelines were reviewed, and experts were consulted. Inclusion criteria were: depression had to be measured at least twice during the study with qualified instruments and the chronic somatic illness had to be assessed by self-report or by a medical professional. Information on depressive recurrence was extracted and additionally risk ratios of recurrence were calculated.

**Results:**

The search generated four articles that fulfilled our inclusion criteria. These studies showed no differences in recurrence over one- two- three- and 6.5 years of follow-up for a total of 2010 depressed patients of which 694 patients with a co-morbid chronic somatic illness versus 1316 patients without (Study 1: RR = 0.49, 95% CI, 0.17–1.41 at one year follow-up and RR = 1.37, 95% CI, 0.78–2.41 at two year follow-up; Study 2: RR = 0.94, 95% CI, 0.65–1.36 at two year follow-up; Study 3: RR = 1.15, 95% CI, 0.40–3.27 at one year follow-up; RR = 1.07, 95% CI, 0.48–2.42 at two year follow-up and RR = 0.99, 95% CI,0.55–1.77 at 6.5 years follow-up; Study 4: RR = 1.16, 95% CI, 0.86–1.57 at three year follow-up).

**Conclusion:**

We found no association between a heightened risk for depressive recurrence and co-morbid chronic somatic illnesses. There is a need for more longitudinal studies to justify the current specific treatment advice such as long-term pharmacological maintenance treatment for this presumed “double trouble” group.

## Introduction

Major Depressive Disorder (MDD) has a highly recurrent nature [1] with relapse and recurrence rates rising up to 85% [2]. Recurrence is defined as a new Major Depressive Episode (MDE) after recovery, that is at least six months without meeting full MDE criteria, whereas relapse is the return of a MDE during remission but before recovery (1988, MacArthur Foundation Research Network on the Psychobiology of Depression) [3].

Most frequently used international evidence based clinical practice guidelines i.e. National Institute for Health & Clinical Excellence (NICE) [4], [5], American Psychiatric Association (APA) [6], presume that co-morbid chronic general somatic disorders are associated with poorer outcomes of depression including an increased risk of recurrence (for readability we always refer to recurrence in case of relapse or recurrence).

Depression is more prevalent in people suffering somatic illness, with prevalence rates being two [7] to even three times as high as for people without somatic illness [6], [8]. Depression has been associated with poorer outcomes of somatic illness [5], [8]–[10], in terms of more functional disability, higher care consumption and a lower quality of life [11]. Conversely, the presence of a co-morbid chronic somatic condition is perceived as an ongoing stressor that predisposes patients to depressive episodes [6]. The APA [6] describes “the presence of a chronic general medical disorder” as a risk factor for recurrence of MDD and therefore recommends that “some form of maintenance treatment will be required indefinitely”.

### Objectives

Remarkably, there are no reviews or meta-analyses that examined the effect of co-morbid chronic somatic illness on depressive recurrence. Given the impact of current international clinical guidelines, we aim to systematically review the evidence as to whether somatic illness is a risk factor for recurrences of MDD over a period of at least six months. If possible a meta-analysis will be performed as well (depending on the number of studies and their methodological characteristics).

## Methods

### Inclusion criteria

Inclusion criteria for our review were: (1) longitudinal measurement of the course of depression (2) providing absolute numbers or percentages of recurrence a) diagnosis established with an interview based on state- of the- art depression criteria (e.g. Diagnostic and Statistical Manual of Mental Disorders, DSM-III/III-R/DSM-IV) [12]–[14] or b) with standardized questionnaires that assess depressive symptoms (e.g., Inventory of Depressive Symptomatology, IDS) [15] or -, Hamilton Rating Scale for Depression, HRSD) [16] (3) with a follow-up of at least six months (4) in which data were collected for patients with and without a certain co-morbid chronic somatic illness at the same measurement intervals a) where co-morbid chronic somatic illnesses were assessed either via self-report or b) medical records or c) by a diagnosis of a medical professional. There is great overlap between self-report of somatic illnesses and diagnoses of these illnesses [17], therefore studies that used self-report as a measurement tool were included as well. If treatment effects were studied within a randomized controlled trial without a treatment-as-usual group, these were excluded. We also excluded studies on bipolar disorders. All relevant publications in English, Dutch, Spanish, Polish or German were taken into account. To assess eligibility of articles, one reviewer (GK) made the first selection based on titles. In case of doubt abstracts or full text articles were retrieved for closer reading. Thereafter, two independent reviewers made a selection based on abstracts (GK and WH); further winnowing was performed by two reviewers (GK and CB) based on full text articles. In case of inconsistencies, articles were evaluated again until consensus was reached. The kappa statistic for inter-observer variability was reported for the abstract and full-text selection.

### Literature search

In line with the APA [6] and NICE [4], [5] treatment guidelines we limited the search to chronic somatic illnesses. The chronic somatic illnesses chosen for this systematic review were: heart diseases, gastrointestinal diseases, diabetes mellitus, rheumatoid arthritis, asthma, Human Immunodeficiency Virus (HIV) and neoplasms. The choice of these chronic somatic illnesses was based on them being mentioned in the clinical practice guidelines of the APA [6] and NICE [4] and their high prevalence rates brought up in additional literature [18], [19].

A combination of MeSH-terms and free text words was entered into the search engines PubMed, EMbase and PsycINFO. These were all screened for relevant articles through 4 December 2012. The terms ‘depression or depressive disorder or major depression were combined with heart diseases or gastrointestinal diseases or diabetes mellitus or arthritis, rheumatoid or asthma or HIV or neoplasms and incidence or follow-up studies or prognos* or predict* or course or outcome or relaps* or recur* or remis* or epidemiology. The key words regarding depression outcomes were based on Altman [20] (box 2) and modified by adding relaps*, recur* and remis* to fit our research purpose. Since not all studies might explicitly mention recurrence, even though they studied the impact on for example chronicity, including recurrence, we decided on the above mentioned broad search terms. Additionally, reference lists from included articles, earlier reviews and NICE [4], [5] and APA [6] clinical treatment guidelines were screened for other potentially eligible papers and experts were consulted to identify additional important papers.

### Data extraction and outcomes

The following data were extracted from the included articles: study site, number of participants, their age and gender distribution, information about depression assessment (method and measurement intervals), characteristics of the chronic somatic illnesses (type and assessment method) and the outcome measure (recurrence). Outcomes consisted of the differences in percentages or mean numbers of recurrence during follow-up, if applicable for multiple time intervals, between patients with and without co-morbid chronic somatic illness. Risk ratios (RR) and 95% Confidence Intervals (CI) were calculated by using Review Manager 5.1.

### Quality assessment

To allow for judgments on the quality of the included articles regarding their selection process, design, analyses and outcome measures, a modified version of the Newcastle-Ottowa Quality Assessment Scale for cohort studies was used [21]. This instrument was reviewed by Deeks et al. [22] and described as one of the most usable methods for this type of study [22], [23]. All articles were judged by two reviewers (GK and WH). If information on a quality criterion was not mentioned explicitly in the article, we assigned a question mark.

## Results

### Study selection

**Figure 1:**
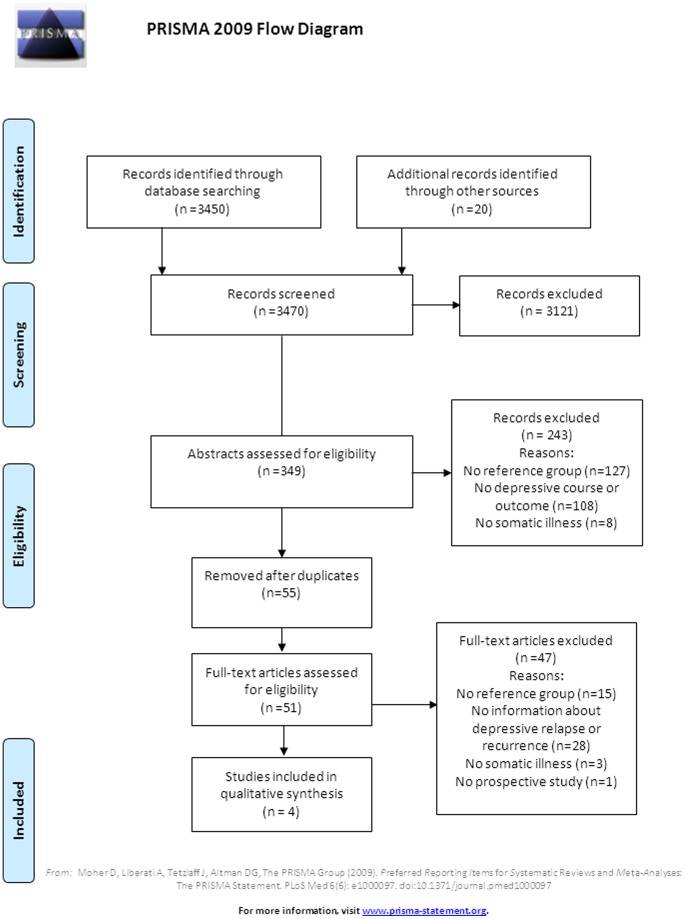
Flow chart of the study selection.

As shown in figure 1, the search engines yielded 3450 articles in total. Twenty records were identified via additional sources including APA guidelines (n = 6), experts (n = 2), reference lists from subsequently included articles (n = 10), and important reviews (n = 2). After screening based on title done by one reviewer (GK), 3121 articles were excluded. Main reasons for exclusion based on title were: studies reported about the influence of depression in a somatic illness population only or about the prevalence of depression only. A total of 349 abstracts subsequently were screened by two independent reviewers (GK and WH). After removal of 55 duplicate titles, eventually 51 full text articles were retrieved for close reading. Detailed information on reasons for exclusion is shown in the flow diagram (figure 1). Finally, four articles about recurrence of depression in patients with and without co-morbid chronic somatic illness were included. The Kappa statistic for the inter-observer variability was 0.91.

### Data extraction from included studies

Data were extracted for the four articles with a total number of 2010 patients (n = 554 in study 1, n = 715 in study 2, n = 54 in study 3, and n = 687 in study 4) yielding information on 694 patients (34.5%) who suffered one or more chronic somatic illness. Table 1 gives an overview of the data extracted from the four studies.

**Table 1:** Data extraction and outcomes of the four included studies.

Authors (publication year)	Number of patients	Age	Gender, female (%)	Depression assessment 1) Baseline 2) Follow-up	Illness assessment	Outcome measures	Results
Wells, Rogers, Burnam & Camp (1993)	554 total (3.8% SI, 96.2% NSI)	-	73.8% (IDDM) 72.8% (non-IDDM)	1) Eight- item depression symptom scale, DSM III criteria 2) Course of depression interview over the phone, based on DIS	Screening and structured somatic history interview	Percentage of patients with more than two depressive spells during follow -up 9.9 years	1 year: SI: 14.9% NSI: 29.3% 2 year: SI: 36.0% NSI: 27.8%
Gerrits, van Oppen, van Marwijk, van der Horst & Penninx (2013)	715 total (44.5% SI 55.5% NSI)	42.1^a^	66%	1)CIDI, DSM IV 2) CIDI, DSM IV and Life Chart Inventory	Self-report	Percentage of patients with remission with recurrence of symptoms (at least three months symptom-free interval)	2 year: SI: 6.0% NSI: 8.0%
Kovacs, Goldston, Obrosky & Drash (1997)	54 total (44.4% SI, 55.6% NSI)	11.2^a^	61.9%	1) ISCA, DSM-III 2) ISCA, DSM-III	Diagnoses at hospital	Recurrence rates: cumulative proportion of developing a new episode of depression after recovery	1 year: SI: 26% NSI: 22% 2 year: SI:30% NSI: 32% 6.5 year: SI: 47% NSI: 47%
Hardeveld, Spijker, De Graaf Nolen & Beekman, 2012	687 total (48.2% SI, 51.8% NSI)	40.7^b^	68.0%	1) CIDI, DSM-III-R 2) CIDI, DSM-III-R retrospectively and prospectively at T1 or T2	Self-report, list of 31 mostly chronic somatic conditions	MDE recurrence between baseline and follow-up (one-or three years) for patients in partial or complete remission for at least six months	SI: 21.1% NSI: 18.3%

*Note,* SI = Somatically Ill; NSI = Non-Somatically Ill; ^a^  =  mean age; ^b^ =  median age; IDDM =  Insulin-Dependent Diabetes Mellitus; DSM III/III-R/IV criteria =  Diagnostic and Statistical Manual of Mental Disorders, third edition, third edition revised; fourth edition; DIS = Diagnostic Interview Schedule; CIDI =  Composite International Diagnostic Interview; ISCA =  Interview schedule for children and adolescents; MDE = Major Depressive Episode.

### Study characteristics

Study 1: A non-experimental longitudinal follow-up study by Wells, Rogers, Burnam, and Camp [24] with adults who received care in one of the following settings: “large group-practice-style health maintenance organizations (HMOs), large multispecialty mixed prepaid and fee-for-service group practices, and small single-specialty group and solo practices”. Patients with either a current depressive disorder or depressive symptoms where included (n = 554 patients). A depressive disorder was defined as meeting DSM-III criteria [12] for a lifetime diagnosis of MDD or dysthymia, an episode of MDD or dysthymia during the past 12 months, with no remission since the onset of the recent episode. Depressive symptoms were present when the cut-off score on a brief depression screener was exceeded [25]. According to the authors (personal communication), patients were asked how many episodes they experienced over follow-up with at least a two month break of depressive symptoms between episodes. The group consisted of 21 (3.8%) patients who suffered current Insulin-Dependent Diabetes Mellitus (IDDM) and 533 (96.2%) without IDDM. Two other somatic illnesses and their relation with depressive recurrence were studied by Wells et al. [24] however we only used the information on the IDDM group while this was the only current chronic somatic illness mentioned.

Study 2: The study of Gerrits, van Oppen, van Marwijk, van der Horst and Penninx [26] is a cohort study (ages 18–65 years) that consisted of 1209 adult participants with a current depression or anxiety diagnosis as assessed by using the Composite International Diagnostic Interview (CIDI) [27], based on DSM-IV criteria [14], followed across two years. Only the information on depression recurrence was used (received through personal communication with the authors) which resulted in data on 715 patients with depression. A total of 318 patients (44.5%) had one or more of the following chronic somatic diseases: cardio-metabolic, respiratory, musculoskeletal, digestive, neurological, endocrine and cancer.

Study 3: Kovacs, Obrosky, Goldston, and Bonar [28] included 54 children (ages 8–13 years) in their longitudinal follow-up study. There were 24 children (44.4%) with current IDDM and 30 children (55.6%) with no other somatic disorders from the same children’s hospital. MDD was assessed with the semi structured Interview Schedule for Children and Adolescents (ISCA) [29]. The control subjects without somatic co-morbidity and a first MDE were balanced for age of onset of first MDD, other co-morbidities and basic characteristics. Of the 24 participants with co-morbid IDDM, six (25%) already suffered from a major psychiatric disorder (i.e. n = 4 with anxiety disorder, n = 1 conduct disorder and n = 1 with functional enuresis). The follow-up period was almost ten years (mean of 9.9 years). Recovery was defined as not fulfilling criteria for MDE, i.e. the absence of symptoms or the presence of few subclinical symptoms, and persistence of this state for at least two months. Of the participants with IDDM, 21 (87.5%) recovered from a first episode as for 29 (96.7%) of the control group.

Study 4: Hardeveld, Spijker, De Graaf, Nolen and Beekman [30] included 687 patients from the general population (ages between 18–64 years). Of the total group, 331 patients (48.2%) had a somatic disease, which was assessed by a questionnaire including 31, mostly, chronic somatic illnesses during the past 12 months. To be included in the study, patients had to be in partial or complete remission of MDD and/or dysthymia for at least six months and the amount of months of being in remission could differ between patients. Remission was defined as: not meeting the full MDE criteria and was assessed at baseline by the computerized version of the CIDI [27]. MDE recurrence was assessed between baseline and three year follow-up. Recurrence was defined as the return of a MDE after partial or complete remission of at least six months. The authors provided us with the absolute numbers of MDE recurrence for patients with and without a somatic illness.

Information on the quality criteria for all four included studies is presented in table 2.

**Table 2:** Quality assessment of the included studies according to a modified version of the Newcastle-Ottowa Quality Assessment Scale for cohort studies.

Domain	Wells, Rogers, Burnam & Camp (1993)	Gerrits, van Oppen, van Marwijk, van der Horst & Penninx (2013)	Kovacs, Goldston, Obrosky & Drash (1997)	Hardeveld, Spijker, De Graaf, Nolen & Beekman (2012)
Representativeness of cohort	*	*	**-**	*
Selection of the non- exposed cohort	*	*	**?**	*
Ascertainment of exposure	**-**	*	*	**-**
Comparability of groups with and without somatic illness on basis of design or analysis	*	*	*	*
Assessment of depression at baseline (blinding demanded)	**-**	**?**	**-**	**-**
Assessment of depression at follow-up (blinding demanded)	**-**	**-**	**-**	**-**
Follow-up at least 6 months?	*	*	*	*
Adequacy of follow-up of cohorts	**?**	**?**	**?**	**-**

*Note,* * = rated as meeting the quality criterion, - = rated as not meeting the quality criterion; ? = no information about quality criterion.

### Risk Ratios

**Figure 2:**
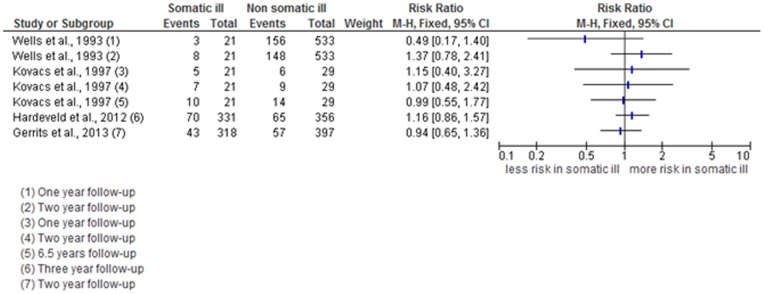
Forest plot of the risk ratios of depressive recurrences with co-morbid chronic somatic illness for all four included studies at their different measurement intervals.

In figure 2 an overview of the number of recurrences in both the patients with and without somatic illness are presented and the RR's of the four studies at all the follow-up intervals are shown in a forest plot.

As shown in table 1 the reported recurrence rates in Wells et al. [24] are lower at the one year follow-up but higher at the two year follow-up in the current IDDM group compared to the group without IDDM (14.9% vs. 29.3% at one year follow-up; 36.0% vs. 27.8% at two year follow-up*).* The calculated RR at one year follow-up is far below one, which might indicate less risk for recurrence of depression in the IDDM patients (n = 21) in comparison to the non-IDDM patients (n = 533). However at two year follow-up there is a shift to a somewhat higher than one RR, indicating more risk for depressive recurrence in the IDDM patients compared to the non-IDDM patients. In the IDDM group, the calculated RR's at one-and two year follow-up are based on recurrences of three and eight patients respectively. Also, the fact that the 95% CI contains the score of one in both cases leads us to conclude that there is no higher risk for two or more depressive episodes for patients with co-morbid IDDM compared to patients without co-morbid IDDM (RR = 0.49, 95% CI, 0.17–1.40; RR = 1.37, 95% CI, 0.78–2.41).

Among the 715 participants in the study by Gerrits et al. [26], 57 (8.0%) of the participants who did not have a co-morbid chronic somatic illness experienced a recurrence after remission versus 43 participants (6.0%) who did have one or more co-morbid chronic somatic illnesses. The RR of having one or more chronic somatic illnesses was around one (RR = 0.94, 95% CI, 0.65–1.36) meaning that in this sample there was no higher risk of recurrence after remission for patients with co-morbid chronic somatic illnesses.

Kovacs et al. [28] found similar recurrence rates in the illness group at one, -two -and 6.5 years of follow-up (respectively: 26% in the somatic illness group versus 22% in the control group; 30% in the somatic ill versus 32% in the control condition; 47% in both the somatically ill and the control condition). RR's range from 0.99–1.15, which again is around one and indicates that the presence of co-morbid IDDM therefore did not seem to heighten the risk of recurrence in this group at one-, two-, and 6.5 year follow-up respectively (RR = 1.15, 95% CI, 0.40–3.27; RR = 1.07, 95% CI, 0.48–2.42; RR = 0.99, 95% CI, 0.55–1.77).

In the study of Hardeveld et al. [30] 135 patients (19.7%) experienced a recurrence. The median time to recurrence was six years (SD = 5.5). MDE recurrence rate was 21.1% for patients with a somatic illness compared to 18.3% for patients without a somatic illness. The RR of MDE recurrence was close to one (RR = 1.16, 95% CI, 0.86–1.57). This indicates that there is no significant heightened risk for a depressive recurrence, between baseline and three years of follow-up, for patients with a co-morbid somatic illness in comparison to patients without a co-morbid somatic illness.

## Discussion

The aim of this systematic review was to determine whether having a co-morbid chronic somatic illness in MDD predicts a greater risk of depressive recurrence. Only four studies examined recurrence in patients with- and without co-morbid chronic somatic illnesses. Surprisingly, there was no indication that co-morbid somatic illness was associated with a greater risk for recurrence.

### Strengths and limitations

We applied broad search terms and inclusion criteria, and acquired studies through three important databases, reference lists, guidelines and experts in the field. This led to the identification of a large number of articles that were reviewed in a systematic fashion by multiple reviewers. Most studies failed to meet the inclusion criteria, mainly because they did not include a reference group without a somatic illness. Rather, these studies presented results of depression and its course within a specific disease group only. Presence of a reference group is crucial in order to draw conclusions on the risk ratio for recurrence that is associated with co-morbid somatic illness.

The included studies differed to a large extent in their methodological characteristics. Therefore it was not appropriate to do a meta-analysis and calculate a pooled risk ratio. The included studies had methodological problems; the quality assessment shows some of these limitations. Whereas baseline depression was defined by DSM criteria in all four studies that was not the case with respect to depressive recurrence at follow-up in the study by Wells et al. [24]. Wells et al. [24] assessed the number of patients with more than two depressive episodes during follow-up. Episodes were defined as periods of depression separated by at least two months of intervening remission as reported by the patient; no DSM criteria were applied to each separate episode. This is not in accordance with the other three studies that did identify each separate episode of depression by applying DSM criteria. Additionally, these episodes cannot be directly compared to MDE recurrences as reported in Gerrits et al. [26], Kovacs et al. [28] and Hardeveld et al. [30]. On the other hand, since Wells et al. [24] reported exclusively more than two episodes during follow-up, the actual rate of recurrences could have been underestimated. Gerrits et al. [26] used a life chart assessment to minimize the risk of missing possible recurrences [31]. Depressive symptomatology fluctuates over time (1) and can be easily overlooked if assessed only at fixed time points, like they did in three out of the four studies [24], [28], [30]. In addition, there are several differences between the studies that further complicated drawing firm conclusions on the overall effect of somatic illness on recurrence rates, such as, different follow-up times, received treatment, type and assessment method of somatic illnesses, depressive symptomatology at baseline, unequal sizes of somatic illness and reference groups (the most unequal sample had 21 somatic ill patients versus 533 patients without somatic illness in the study of Wells et al.) [24]. Also there were differences in the choice and sizes of the reference groups (533, 397, 30 and 356 patients).

### Implications

Current clinical guidelines, such as the APA [6] and the NICE [4], [5], identified the group of MDD patients with co-morbid chronic somatic illness as a “double trouble” group with poor prognosis. Prolonged pharmacological maintenance treatment has been recommended for these patients if they were treated with antidepressants during the acute phase of their depressive episode. Additional preventive psychological treatment was recommended for those who received psychotherapy during acute treatment. However, our review suggests that the number of studies that could provide such evidence is very small and that those few studies that are available provide no indication of any elevated risk of recurrence among depressed patients with co-morbid somatic illness. We therefore call for additional longitudinal studies about the impact of co-morbid chronic somatic illness on the course of depression. Apart from recurrence, other outcomes also are potentially relevant, such as the quality of life, severity and duration of episodes, hospitalization, sick leave from work and persistence of depression. An interesting question might be whether the prognosis of depression could deteriorate when somatic illness progresses and if so, whether treatment of the somatic illness can counter this process. Additionally, we need to study whether maintenance pharmacotherapy's, as well as psychological interventions, are (equally) effective for this presumed high risk group regarding recurrence.
